# Guesstimate of thymoquinone diversity in *Nigella sativa* L. genotypes and elite varieties collected from Indian states using HPTLC technique

**DOI:** 10.1515/biol-2022-0536

**Published:** 2023-02-07

**Authors:** Y. Ravi, Irene P. Vethamoni, Shailendra N. Saxena, S. Velmurugan, V. P. Santanakrishnan, M. Raveendran, Himanshu Bariya, Mistry Harsh

**Affiliations:** Department of Spices, Plantation, Medicinal and Aromatic Crops, Horticulture College and Research Institute, Tamil Nadu Agricultural University, Coimbatore, 641003, India; Division of Crop Improvement, Indian Council of Agricultural Research, National Research Centre on Seed Spices, Ajmer, Rajasthan, 305206, India; Department of Spices and Plantation crops, Horticulture College and Research Institute, Tamil Nadu Agricultural University, Coimbatore, 641003, India; Department of Biochemistry, Centre for Plant Molecular Biology and Biotechnology, Tamil Nadu Agricultural University, Coimbatore, 641003, India; Department of Bio-Technology, Hemchandracharya North Gujarat University, Patan, Gujarat 384265, India

**Keywords:** DIVA-GIS, genotypes, HPTLC, phytochemical variation, thymoquinone

## Abstract

Thymoquinone is a valuable metabolite derived from the *Nigella sativa* L. seeds and has a variety of therapeutic properties. Thymoquinone was estimated using *n*-hexane:ethyl acetate (8:2, v/v) green solvent system and computed at a wavelength of 254 nm using the high-performance thin-layer chromatography densitometry method in distinct varieties and genotypes congregated from different geographical regions. Genotype Ajmer Nigella-13 has the paramount thymoquinone content (247.60 µg/100 mg seed) followed by Ajmer Nigella 19 (244.5 µg/100 mg seed), while the lowest amount of thymoquinone was recorded in the genotype Ajmer Nigella-6 (42.88 µg/100 mg seed). The hierarchical cluster analysis found that the collected genotypes and elite varieties were classified into four broad clusters, and the identified chemotypes with elevated thymoquinone proportion were positioned in cluster D. Significant genotypic variation in thymoquinone content is available, that can be used in exploiting pharmaceutical applications of *N. sativa* L. as well as a breeding programme for specific metabolite improvement perspective.

## 1 Introduction

Natural plant products are usually accepted in the pharmacy sector for their structural diversity and a wide variety of pharmacological actions [1]. The study of phytoconstituents is associated with the large diversity of organic chemicals that plants produce and accumulate, as well as their structures, biosynthesis, circulation, metabolism, natural distribution, and biological function [2]. The use of high-performance thin-layer chromatography (HPTLC) to determine the principal active components of medicinal plants makes perfect sense for the expansion of chromatographic fingerprints. The isolation and magnification are significantly improved, and the results are far more stable and reliable than thin-layer chromatography (TLC) [3]. It has the main advantage of *in situ* descriptive and analytical evaluations using scanning densitometry when combined with digital scanning profiling [4]. Furthermore, the vivid pictorial HPTLC image offers additional, understandable apparent colour, and/or fluorescence parameters for parallel evaluation on the same plate [5]. It also demonstrated improved dissociation of individual secondary metabolites. India, as a mega biodiversity nation, has a high species rich in medicinal plants; despite this, a large number of various medicinal plants have yet to be studied phytochemically, with a focus on secondary metabolites of therapeutic relevance [6].

Nigella or Kalonji (*Nigella sativa* L.) is an erect, herbaceous annual plant, belonging to the plant family Ranunculaceae under the genus nigella [7]. The genus name is derived from the Latin word *niger*, which means “black,” to the colour of the seeds [8]. It is commercially cultivated in India and it is native to the areas of Southern Europe, North Africa, and Southwest Asia, and it is grown in many nations in the Middle East Mediterranean region, as well as in India, Pakistan, Syria, Turkey, and Saudi Arabia [7]. Table 1 contains a detailed plant profile of nigella. Most pharmacological properties of the whole seeds of nigella or their extracts are mainly attributed to its volatile oil, of which thymoquinone (2-isopropyl-5-methyl-1,4-benzoquinone), about 27–57%, is the most abundant component [9]. Thymoquinone has been shown in the studies to have antioxidant [10,11], cardioprotective [12], neuroprotective [13], hepatoprotective [14], anti-inflammatory [15], antimutagenic [16], and antiproliferative [17,18] properties. Further, the previous research has shown that pure thymoquinone and nigella concentrate boost enzyme-mediated DNA breakage [19]; as a result, thymoquinone, like several other nutritional phytochemicals, is a topoisomerase II toxin with anticancer and anti-inflammatory properties [20]. It has been reported to be faintly soluble in water, soluble in methanol and dimethyl sulphoxide, and readily soluble in a number of organic solvents including isopropanol, 1-butanol, 2-butanol, ethyl acetate, carbitol, and polyethylene glycol-400 [14].

**Table 1:** Systematic classification of nigella plant [8]

Grouping	Description
Plant kingdom	Plantae
Subkingdom	Viridiplantae
Superdivision	Embryophyta
Division	Tracheophyta
Subdivision	Supermatophytina
Class	Magnoliopsida
Superorder	Ranunculanae
Order	Ranunculales
Family	Ranunculaceae
Genus	Nigella
Species	Sativa

Genetic variability is an important factor for varying extents of metabolite obtained in different varieties of crop plants [21]. Several medicinal plants have been studied regarding the variability in the amount of active phytochemicals in different varieties of the same plant grown in different regions [22,23]. Due to the significant difference in environmental circumstances at various sites as well as genetic variation of the material, medicine quality is inconsistent throughout the industry. Additionally, advanced analytical methods for determining thymoquinone have enabled it to empirically explore the environmental, genotypic, and ontogenetic variability in *N. sativa* L. and there are few publications on the level of genotypic variability in cultivated nigella genotypes with regard to thymoquinone content. The study was undertaken with the objective of identifying an appropriate genotype with enhanced metabolite content for industrial and pharmaceutical applications using a reliable approach via., HPTLC analysis. This is the principal documentation for the evaluation and documentation of thymoquinone diversity in the released varieties and genotypes collected from different parts of India.

## 2 Materials and methods

### 2.1 Plant material

Five national released varieties and 35 unique collections of *N. sativa* L. gathered across India were evaluated for the presence and estimation of thymoquinone, a highly valued metabolite present in seed and its diversity. The test samples were analysed and chromatograms were acquired under the same conditions along with standard thymoquinone. The area of the peak corresponding to the *Rf* (retention factor) value of the thymoquinone standard was quantified, and the amount present was computed using the regression equation from the calibration plot.

**Figure 1:**
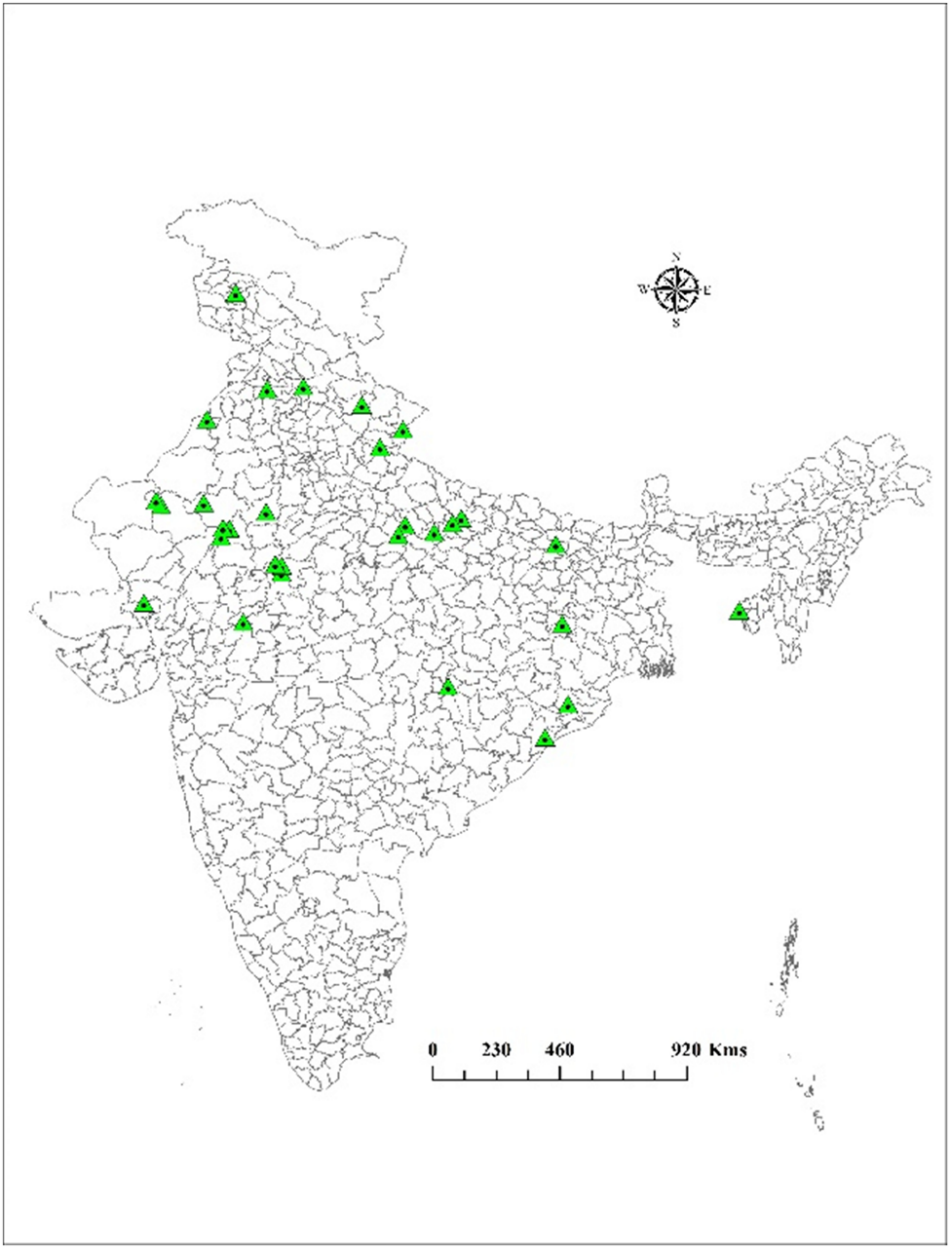
DIVA-GIS mapping of *N. sativa* L. genotypes and elite varieties collection sites used in the study.

To explore the genetic diversity of *N. sativa* L. present in India, 40 accessions were collected from different agro-climatic areas of India based on the spatial range and morphological diversity (Table 3). The augmented germplasm comprised 14 Indian states viz., Rajasthan (17), Uttar Pradesh (6), Uttarakhand (4), Odisha (2), Tripura (2), Madhya Pradesh (1), Gujarat (1), Punjab (1), Himachal Pradesh (1), Jammu and Kashmir (1), Jharkhand (2), Bihar (1), and Chhattisgarh (1) as shown in Figure 1. The field experiment was carried out at farm field of ICAR NRCSS, Ajmer, located at the latitude of 26°27′0″N and 74°38′0″E longitude having 460 m mean sea level (MSL) altitude by adopting recommended package of practices to screen nigella varieties and accessions for thymoquinone metabolite. After 130 days of sowing, the seeds were harvested, shade dried, and ground into powder for analysis of thymoquinone using a previously standardized HPTLC method [23]. DIVA-GIS version 7.1.6, free downloadable software was utilized for mapping the diversity of *N. sativa* L. germplasm accessions used in this study. A geographical positioning system (Garmin 12) was used to record the geographical coordinates (latitude and longitude) of the collecting sites.

### 2.2 Extraction of raw drugs from seeds

Accurately weighed 10 g seeds were powdered in Malavasi mill (Bologna, Italy) taking care to avoid overheating and the particle sizes were in the range of 250–425 µm. Methanol, the extraction solvent at a ratio of 4:1 was then poured on top of the seed material and soaked for overnight period. After 12 h of soaking the material was filtered through Whatman filter paper and the filtered material crude extract mixture was centrifuged at 2,500 rpm for 6 min. The collected extract was subjected to HPTLC analysis. This method is suitable for extraction of bioactive constituents that are readily soluble. In addition, it is an appropriate method for the preparation of fresh extract before use [24].

### 2.3 Metabolite identification

**Figure 2:**
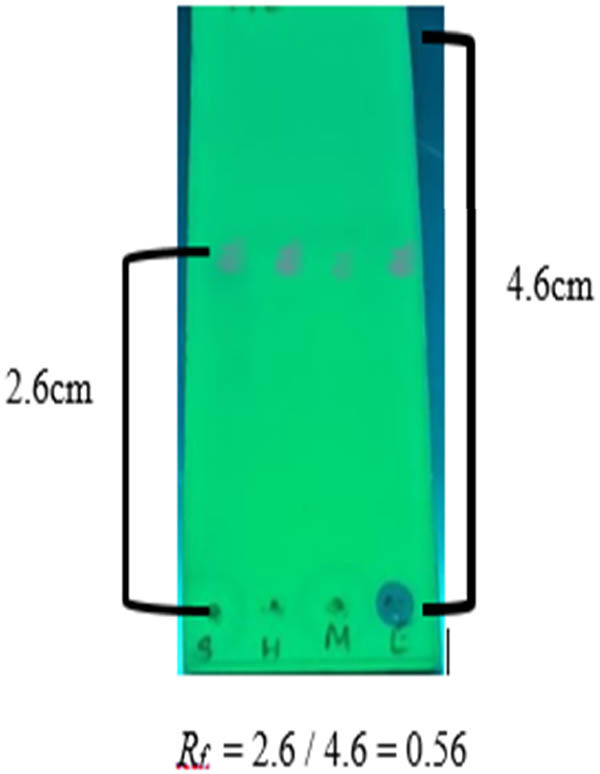
TLC to identify thymoquinone under UV light.

TLC plates (Merck, Darmstadt, Germany) with the specifications of silica gel 60G, with dimensions of 4.5 mm × 10 mm, was used to identify thymoquinone as suggested by Basha et al. [25]. The development system was made up of *n*-hexane:ethyl acetate (8:2 v/v), which produced a strong and well-defined band for the metabolite and the identity was validated by comparing the bands of standard thymoquinone with those of studied sample extracts, as well as the *Rf* (0.56) of the reference with that of the sample (Figure 2).

### 2.4 Chromatographic conditions for identification of thymoquinone

HPTLC densitometry analysis was performed on 10 cm × 20 cm aluminium-backed plates coated with 0.2 mm layers of silica gel 60 F254 (E-Merck, Germany). Using a Camag Automatic TLC Sampler 4 sample applicator (Switzerland) equipped with a Camag microlitre syringe, samples were casted to the TLC plates as 6 mm bands. The application rate was fixed constant at 150 nL/s. Linear ascending development of the plates to a distance of 80 mm was carried out in a Camag Automatic Developing Chamber 2 previously saturated with mobile phase vapour for 30 min at 22°C using *n*-hexane:ethyl acetate 8:2 (percent, v/v) as mobile phase. The plates were scanned at 259 nm using a Camag TLC scanner in absorbance mode with a deuterium light after development. The scanning speed was 20 mm/s and the slit dimensions were 4.00 mm × 0.45 mm [26].

### 2.5 Method and calibration curve development

**Figure 3:**
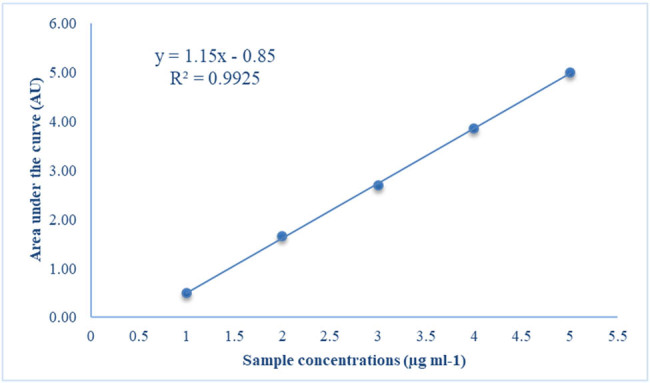
Standard thymoquinone calibration curve.

The mobile phase composition was modified in order to develop an appropriate and accurate design to address the HPTLC method for thymoquinone analysis. The mobile phase *n*-hexane:ethyl acetate 8:2 (percent, v/v) revealed a vivid, symmetrical, and highly defined peak at *Rf* = (0.91 ± 0.02). Maximum absorbance was seen in the bands’ UV spectra at approximately 254 nm. Thymoquinone stock solution (1.0 mg/mL concentration) was made by dissolving 1.0 mg of thymoquinone in HPLC grade methanol and diluting to a final volume of 1 mL, then sonicated at 37°C for 10 min [27]. Before HPLC analysis, all dilutions were made stepwise through the main stock in methanol and subsequently filtered through a 0.22 µm membrane filter. In methanol, calibration curve of thymoquinone from five reference points (1, 2.5, 5.0, 7.5, and 10 µg/mL) as depicted in Figure 3. The calibration curves were plotted using thymoquinone (1.0 mg/mL) linear least square regression on analyses concentration versus peak area.

The standard deviation (SD) method was used to calculate the limit of detection (LOD) and limit of quantitation (LOQ). They were calculated using the following equations premised on the slope of the calibration (*S*) curve and the SD of the blank sample:

\text{LOD}=3.3\times \text{SD/}S.]

\text{LOQ}=\text{10 times}\times \text{SD/}S.]

LOD and LOQ found in the study are 0.77 and 2.34 ng/spot (Table 2), for detection and quantification of thymoquinone effectively from the studied samples [28].

**Table 2:** Linear regression data for the calibration curve of thymoquinone (*n* = 40)

**Set**	**Description**
Linearity range (µL/spot)	0.50–5.00
Regression equation	*y* = 1.15*x* − 0.85
Correlation coefficient	0.9925
LOD	0.77 ng/spot
Limit of quantification	2.34 ng/spot

### 2.6 Data analysis

To investigate the variability, the 40 genotypes and elite varieties were subjected to hierarchical cluster analysis using the ward method based on the thymoquinone content contained in seed extract [29], and Microsoft Excel 2016 was used for the calculation of calibration curved, fitting regression equation, and plotting.

## 3 Results

### 3.1 Estimation of thymoquione metabolite

**Figure 4:**
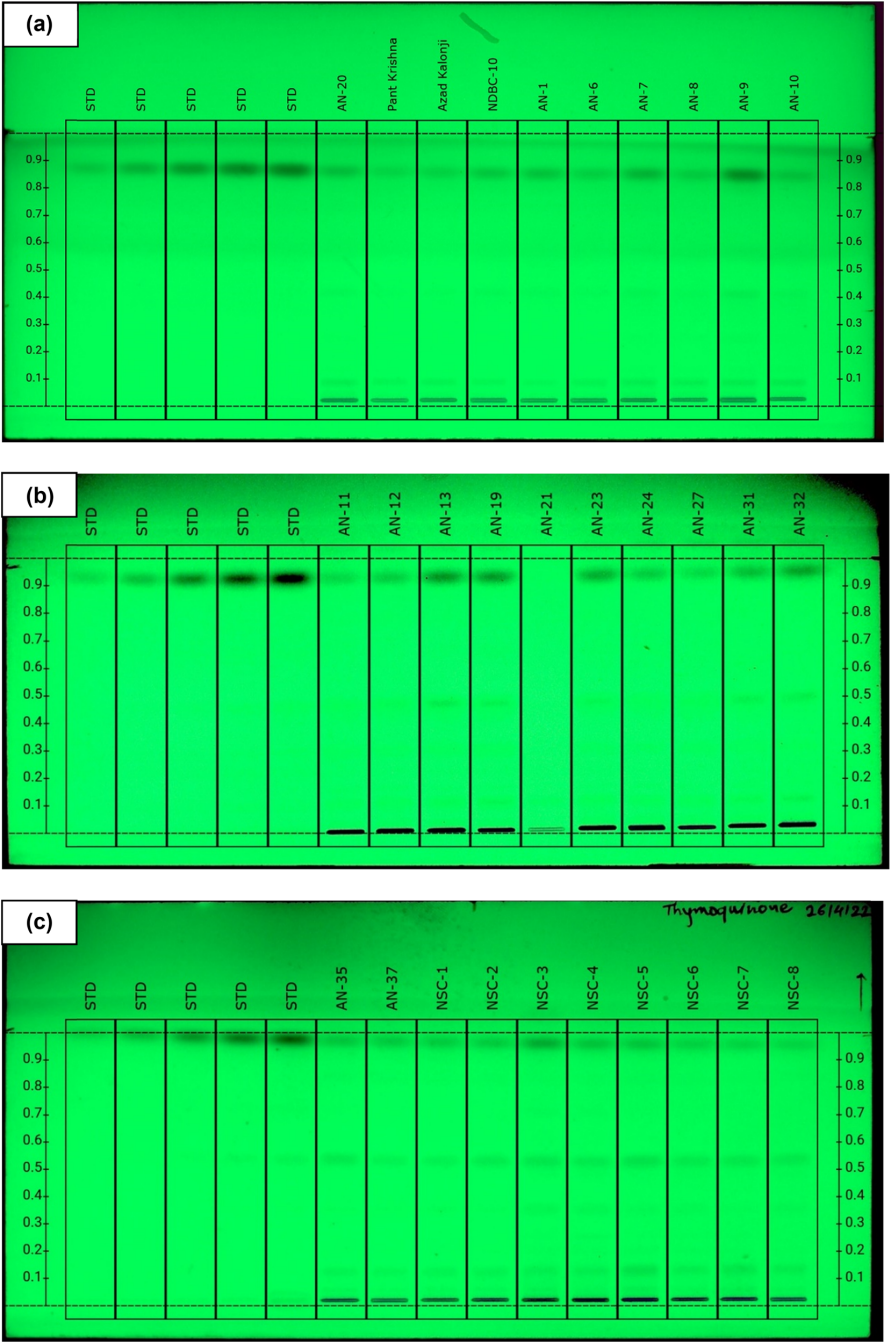

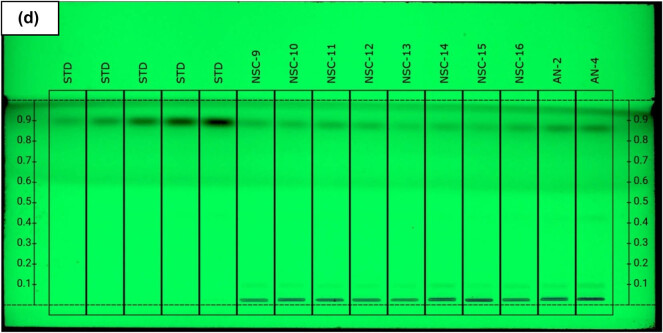
HPTLC separation of *N. sativa* L. genotypes and elite varieties seed extract in mobile phase n-hexane:ethyl acetate (8:2, v/v) observed under UV illumination at 254 nm; tracks STD - standard at five different concentrations. (a) Thymoquinone standard concentration and in studied genotypes, (b) thymoquinone standard concentration and in studied genotypes, (c) thymoquinone standard concentration and in studied genotypes, (d) thymoquinone standard concentration and in studied genotypes.

**Figure 5:**
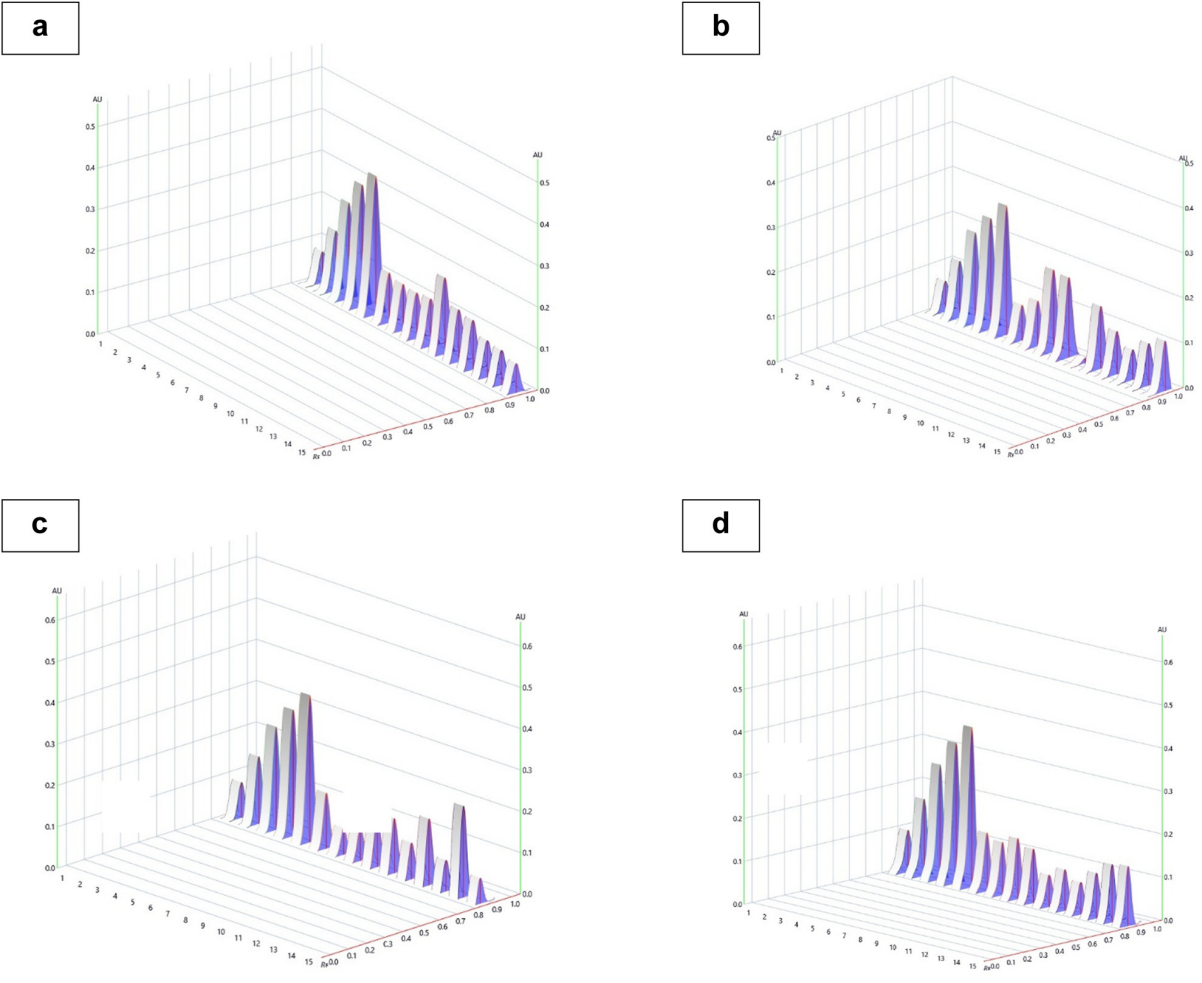
HPTLC integrated peaks of *N. sativa* L. genotypes and elite varieties in mobile phase *n*-hexane:ethyl acetate (8:2, v/v) observed under UV illumination at 254 nm. (a–d) Thymoquinone standard concentration and in studied genotypes.

Thymoquinone peaks in studied varieties and accessions were identified by comparing their single spot at *Rf* = 0.91 values to those obtained by standard chromatogram. The thymoquinone content was assessed using the linear regression equation and concentration. Table 2 shows the estimated composition of thymoquinone in the examined varieties and accessions, while Figures 2 and 3 show the chromatograms and the integrated peaks of the studied varieties and accessions. The thymoquinone content varied among the genotypes and ranged from 42.88 µg/100 mg to 247.60 µg/100 mg (Figures 4a–d and 5a–d; Table 3). The highest amount of thymoquinone has been recorded in the genotype Ajmer Nigella-30 (247.60 µg/100 g seed) followed by Ajmer Nigella-13 (244.50 µg/100 mg seed); whereas, the lowest amount of thymoquinone has been recorded in the genotype Ajmer Nigella-6 (42.88 µg/100 mg seed). Furthermore, the genotypes Ajmer Nigella-21 and Ajmer Nigella-27 (Figures 4b and 5b) have not detected the thymoquinone content, for the reason that the amount of metabolite might have been lesser than the standard concentration.

**Table 3:** Thymoquinone content in *N. sativa* L. genotypes and elite varieties from different geographical regions of India

**Sl. No.**	**Sample**	***Rf***	**Height**	**Area**	**Thymoquinone**
1.	Standard 1 µg/mL	0.934	0.0680	0.00354	0.50 µg/mL
	Standard 2.5 µg/mL	0.927	0.1263	0.00691	1.25 µg/mL
	Standard 5.0 µg/mL	0.927	0.2029	0.01136	2.50 µg/mL
	Standard 7.50 µg/mL	0.929	0.2472	0.01399	3.75 µg/mL
	Standard 10.00 µg/mL	0.929	0.2472	0.01399	5.0 µg/mL
2.	Ajmer Nigella-20	0.877	0.1341	0.00576	141.70 µg/100 mg
3.	Pant Krishna	0.876	0.0638	0.00261	64.31 µg/100 mg
4.	Azad Kalonji	0.871	0.0794	0.00338	83.13 µg/100 mg
5.	NDBC-10	0.868	0.1204	0.00499	122.80 µg/100 mg
6.	Ajmer Nigella-1	0.865	0.1274	0.00518	127.40 µg/100 mg
7.	Ajmer Nigella-6	0.863	0.0838	0.00325	42.88 µg/100 mg
8.	Ajmer Nigella-7	0.861	0.1524	0.00590	80.00 µg/100 mg
9.	Ajmer Nigella-8	0.858	0.0701	0.00247	145.20 µg/100 mg
10.	Ajmer Nigella-9	0.858	0.2013	0.00716	60.72 µg/100 mg
11.	Ajmer Nigella-10	0.853	0.0558	0.00174	176.3 µg/100 mg
12.	Ajmer Nigella-11	0.932	0.0792	0.00433	43.95 µg/100 mg
13.	Ajmer Nigella-12	0.934	0.1013	0.00557	87.72 µg/100 mg
14.	Ajmer Nigella-13	0.937	0.1829	0.01011	247.60 µg/100 mg
15.	Ajmer Nigella-19	0.937	0.01785	0.01002	244.50 µg/100 mg
16.	Ajmer Nigella-21	0.942	0.0111	0.00053	Not detected
17.	Ajmer Nigella-23	0.944	0.133	0.00749	155.20 µg/100 mg
18.	Ajmer Nigella-24	0.948	0.0957	0.00505	69.21 µg/100 mg
19.	Ajmer Nigella-27	0.952	0.0673	0.00345	Not detected
20.	Ajmer Nigella-31	0.955	0.0937	0.00465	55.32 µg/100 mg
21.	Ajmer Nigella-32	0.961	0.1116	0.00523	75.61 µg/100 mg
22.	Ajmer Nigella-35	0.974	0.1207	0.00570	140.70 µg/100 mg
23.	Ajmer Nigella-37	0.973	0.1124	0.00523	129.10 µg/100 mg
24.	*Nigella sativa* collection-1	0.969	0.1100	0.00515	127.10 µg/100 mg
25.	*Nigella sativa* collection-2	0.966	0.1148	0.00533	131.70 µg/100 mg
26.	*Nigella sativa* collection-3	0.966	0.1840	0.00866	214.00 µg/100 mg
27.	*Nigella sativa* collection-4	0.963	0.1262	0.00572	141.40 µg/100 mg
28.	*Nigella sativa* collection-5	0.963	0.1192	0.00538	133.00 µg/100 mg
29.	*Nigella sativa* collection-6	0.961	0.0889	0.00392	96.64 µg/100 mg
30.	*Nigella sativa* collection-7	0.961	0.0844	0.00367	90.64 µg/100 mg
31.	*Nigella sativa* collection-8	0.963	0.0708	0.00300	74.18 µg/100 mg
32.	*Nigella sativa* collection-9	0.882	0.1364	0.00695	144.40 µg/100 mg
33.	*Nigella sativa* collection-10	0.879	0.1240	0.00622	125.80 µg/100 mg
34.	*Nigella sativa* collection-11	0.876	0.1415	0.00688	139.10 µg/100 mg
35.	*Nigella sativa* collection-12	0.873	0.1262	0.00614	124.20 µg/100 mg
36.	*Nigella sativa* collection-13	0.871	0.0745	0.00373	75.38 µg/100 mg
37.	*Nigella sativa* collection-14	0.869	0.0959	0.00462	93.34 µg/100 mg
38.	*Nigella sativa* collection-15	0.869	0.0755	0.00367	74.22 µg/100 mg
39.	*Nigella sativa* collection-16	0.866	0.1059	0.00494	99.77 µg/100 mg
40.	Ajmer Nigella-2	0.865	0.1349	0.00607	122.70 µg/100 mg
41.	Ajmer Nigella-4	0.865	0.1394	0.00618	124.90 µg/100 mg

### 3.2 Diversity analysis among genotypes and elite varieties from different geographical regions of India

**Figure 6:**
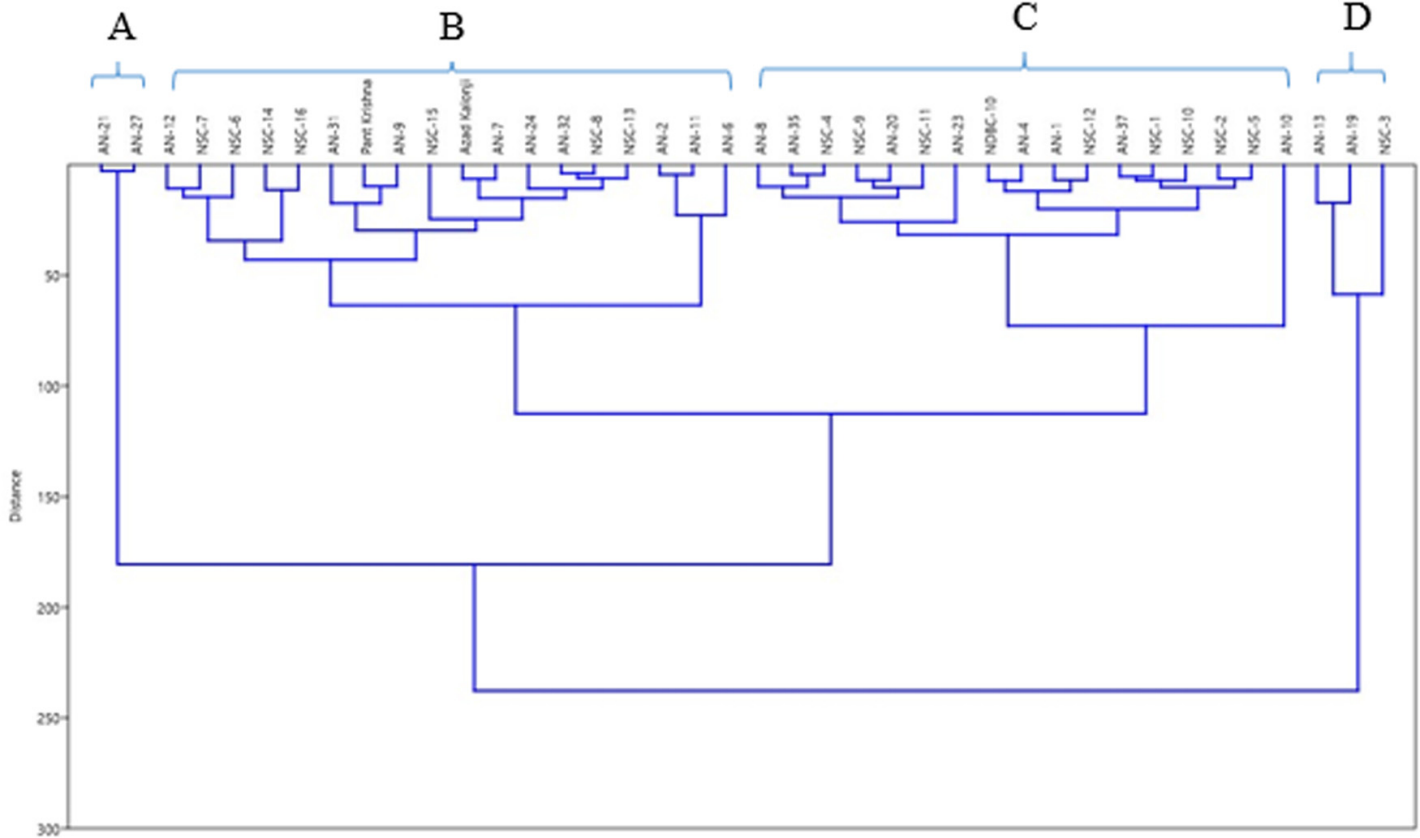
Hierarchical cluster analysis showing the thymoquinone relationship among nigella (*N. sativa* L.) genotypes and elite varieties from different locations in India. (a–d) Different clusters formed among the studied genotypes.

The observed thymoquinone compositional diversity in genotypes and elite varieties of nigella aromatic oil obtained from different locations were statistically validated. A total of 40 genotypes and elite varieties were assessed using hierarchical cluster analysis. Based on the unweighted pair group method with arithmetic mean agglomerative hierarchical clustering the resultant dendrogram established four broad and unique clusters based on the metabolite composition from the seed extract of different genotypes and varieties, namely A, B, C, and D (Figure 6). Cluster A (AN-21 and AN-27) contains genotypes that lack the thymoquinone metabolite. Major Cluster B was separated into four sections (B1, B2, B3 and B4), while Major Cluster C was divided into two sections (C1 and C2). The aromatic oil obtained from the genotypes (AN-13, AN-19, and NSC-03) was high in thymoquinone, according to the chemical composition and cluster analysis.

## 4 Discussion

### 4.1 Quantification of thymoquinone

Nigella has been explored and utilized by millions of people for millennia as one of the most investigated and widely used natural products. Both the seed and its oil have garnered a lot of prominence due to their widespread efficient medicinal ability to cure a variety of ailments. Because of the wide spectrum of biological activity of nigella seed extracts, oils, and isolated chemicals, many studies have been conducted on the multifarious therapeutic roles of thymoquinone in an array of different diseases, particularly in the last two decades. However, phytochemicals produced by a certain plant type could be the cause of fluctuation in yield, and as a result, composition and yield changed appropriately. The outcome of the study is compared with Foudah et al. [28], who found that the highest thymoquinone concentration was found in Saudi Arabian extract (10.76 ± 1.01 mg/g), followed by Syrian extract (8.09 ± 0.62 mg/g), Indian extract (7.14 ± 0.41 mg/g), and commercial capsules (7.03 ± 0.52 mg/g) and Isik [30] wherein the percentage of thymoquinone measured by HPLC in seeds and seeds’ oil varied between 0.014–0.376 and 0.142–0.619%, respectively. It suggests that seeds are a major source of thymoquinone.

### 4.2 Genetic diversity analysis for metabolite content among genotypes and elite varieties from different geographical regions of India

Thymoquinone is a major phytochemical element of nigella seeds, its standardization and formulation required a reliable and intuitive technique. Further, standardization of herbal suppository formulations in terms of raw ingredient quality, manufacturing procedures, and composition are required to ensure quality and the most suitable amounts of active principles for bio-effectiveness. Many researchers have previously investigated the chemical composition of nigella seed extract or oil, but its diversity in connection to specific metabolites has not been comprehensively investigated [31–33]. So we conducted our investigation, which revealed considerable changes in the chemical constitution of thymoquinone metabolites of nigella seed extract generated in various parts of India. Similar results with respect to the response of genotypes from distinct destinations for chemical constituents were reported in davana [34], which obscured that there were significant differences in the physical properties and chemical composition of davana oils produced in India.

Present study signposts that Ajmer Nigella-13, Ajmer Nigella-17, and *Nigella sativa* collection-03 were the prominent sources of thymoquinone metabolite among the studied varieties and genotypes. Similar findings were found in turmeric by [35] wherein they carried out the curcuminoid profile of released varieties and local types, in which local (check) appeared considerably superior in terms of curcuminoids. The variation in the composition of metabolite might be linked to the varietal genetic makeup with the most significant reaction to the given environment coupled with physiological and biochemical phenomena including varying levels of phytochemicals in the given variety of the crop plant. Thus, the distinct quantity of thymoquinone in different varieties implies varied expression.

## 5 Conclusions

Nigella has been explored and utilized by millions of people for millennia as one of the most investigated and widely used natural products. The HPTLC method is a reliable approach for quantifying active constituents that may be used for quality control and standardization of crude drugs and their formulations. It can be used for large-scale germplasm screening and can save analysis costs and time. The genotypes tested with the highest levels of thymoquinone were identified as Ajmer Nigella-13, Ajmer Nigella-19, and *Nigella sativa* collection-3 by the results. The found genotypes could be grown commercially to meet the rising pharmaceutical industry demand and for metabolite extraction. It is possible to exploit the variations present in *N. sativa* L. for thymoquinone content (42.88–247.60 g/100 mg seed) in trait-specific breeding and further crop improvement programmes with a focus on therapeutic and food applications. This study will benefit the readers by acquainting them with the pharmaceutical significance and further usage of the nigella seeds in daily lifestyle enhancement.
